# Recombinant horseradish peroxidase variants for targeted cancer treatment

**DOI:** 10.1002/cam4.668

**Published:** 2016-03-15

**Authors:** Günther Bonifert, Lisa Folkes, Christoph Gmeiner, Gabi Dachs, Oliver Spadiut

**Affiliations:** ^1^ Research Area Biochemical EngineeringInstitute of Chemical EngineeringVienna University of TechnologyViennaAustria; ^2^ Department of Oncology Oxford Institute for Radiation OncologyUniversity of OxfordNorthwoodMiddlesexU.K.; ^3^ Mackenzie Cancer Research GroupDepartment of PathologyUniversity of OtagoChristchurchNew Zealand

**Keywords:** Antibody directed enzyme prodrug therapy (ADEPT), horseradish peroxidase, indole‐3‐acetic acid, MDA‐MB‐231 breast carcinoma, *Pichia pastoris*, T24 bladder carcinoma

## Abstract

Cancer is a major cause of death. Common chemo‐ and radiation‐therapies damage healthy tissue and cause painful side effects. The enzyme horseradish peroxidase (HRP) has been shown to activate the plant hormone indole‐3‐acetic acid (IAA) to a powerful anticancer agent in *in vitro* studies, but gene directed enzyme prodrug therapy (GDEPT) studies showed ambivalent results. Thus, HRP/IAA in antibody directed enzyme prodrug therapy (ADEPT) was investigated as an alternative. However, this approach has not been intensively studied, since the enzyme preparation from plant describes an undefined mixture of isoenzymes with a heterogenic glycosylation pattern incompatible with the human system. Here, we describe the recombinant production of the two HRP isoenzymes C1A and A2A in a *Pichia pastoris* benchmark strain and a glyco‐engineered strain with a knockout of the *α*‐1,6‐mannosyltransferase (OCH1) responsible for hypermannosylation. We biochemically characterized the enzyme variants, tested them with IAA and applied them on cancer cells. In the absence of H_2_O_2_, HRP C1A turned out to be highly active with IAA, independent of its surface glycosylation. Subsequent *in vitro* cytotoxicity studies with human T24 bladder carcinoma and MDA‐MB‐231 breast carcinoma cells underlined the applicability of recombinant HRP C1A with reduced surface glycoslyation for targeted cancer treatment. Summarizing, this is the first study describing the successful use of recombinantly produced HRP for targeted cancer treatment. Our findings might pave the way for an increased use of the powerful isoenzyme HRP C1A in cancer research in the future.

## Introduction

Cancer is one of the major mortality causes worldwide. Over 14 million new cases and approximately 8 million tumor‐related deaths are counted annually 1. Common treatment methods are surgical intervention, radiation therapy, and chemotherapy. One of the main challenges in antitumor therapy is the targeted delivery of the toxic agent to the tumor without harming healthy tissue. However, current therapeutic approaches still damage healthy tissue and cause painful and unpleasant side effects.

**Figure 1:**
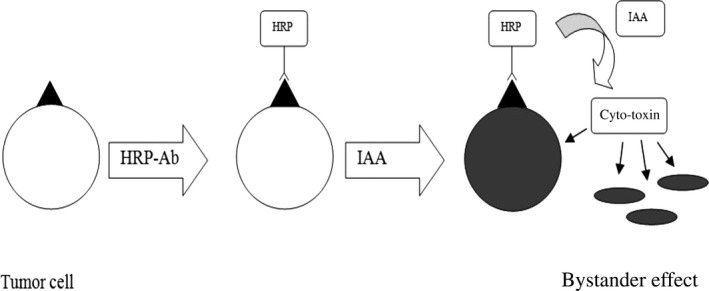
Scheme of antibody directed enzyme prodrug therapy (ADEPT) with indole‐3‐acetic acid (IAA)/ horseradish peroxidase (HRP). HRP is conjugated with a tumor‐specific antibody, which delivers the enzyme directly to the site of the tumor cells. Then, IAA is injected and oxidized to a cyto‐toxin by HRP. Tumor cells are also killed via diffusion of the toxic compound, called bystander effect. Figure adapted from 5.

Potential strategies to overcome this challenge are antibody‐directed enzyme prodrug therapy (ADEPT) and gene‐directed enzyme prodrug therapy (GDEPT) which allow the selective release of a cytotoxic agent from a non‐toxic prodrug at the site of the tumor (e.g., 2, 3). In this respect numerous studies investigated the cytotoxic effect of the prodrug indole‐3‐acetic acid (IAA) after oxidation with the heme‐containing enzyme horseradish peroxidase (HRP, EC 1.11.7.1; 4, 5, 6, 7, 8, 9, 10, 11, 12, 13, 14, 15, 16, 17, 18, 19, 20, 21, 22, 23, 24). In 1998, Folkes et al. showed that the application of IAA/HRP on V79 hamster cells caused cytotoxicity, but neither IAA nor HRP alone were toxic. Furthermore, they found that H_2_O_2_ was not required for oxidation and IAA was not readily oxidized by mammalian peroxidases, making IAA/HRP an attractive couple for targeted cancer therapies. Subsequently, Greco et al. intensively investigated the power of IAA/HRP in GDEPT 4, 7, 8, 11, 12, 13). In 2000, they expressed HRP in human T24 bladder carcinoma cells, which were then incubated for up to 24 h with different concentrations of IAA. The plant hormone was oxidized directly in the tumor cells and after only 2 h of incubation with 0.1 mmol/L IAA and 1.2 *μ*g/mL HRP, 87% of the T24 bladder carcinoma cells, which are known to be resistant to a number of chemotherapeutic drugs 25, lost their clonogenic potential. Furthermore a bystander effect, describing cell kill of nontransfected tumor cells via diffusion of a toxic compound 26, 27, was observed. In more recent studies, Kim et al. demonstrated the cytotoxic effect of IAA/HRP on human G361 melanoma cells 14 and urinary bladder carcinoma cells 15. Another study showed apoptosis of human pancreatic cancer BXPC‐3 cells caused by IAA/HRP 16. After these *in vitro* studies, which impressively demonstrated the power of IAA/HRP to efficiently kill tumor cells, the first *in vivo* study was performed 19. Human nasopharyngeal squamous carcinoma cells stably expressing HRP were grown as xenografts in SCID mice and were treated with IAA and its analog 5Br‐IAA. *In vitro* clonogenic assays indicated that doses of 200 mg/kg IAA and 5Br‐IAA gave a 60 and 45% reduction in cancer cell survival, respectively. However, *in vivo* studies were disappointing since neither prodrug decreased cancer cell survival. The authors speculated that the expression level of HRP in the tumor cells was too low to obtain a sufficient concentration of the desired toxin 19. However, another study successfully demonstrated the *in vivo* efficacy of IAA/HRP on hepatocellular carcinoma cells *in vivo* 23. Speculations arose that the effect of IAA/HRP in GDEPT was cancer cell line dependent. Thus, ADEPT was considered as an alternative to GDEPT. ADEPT employs humanized monoclonal antibodies to deliver HRP directly to the tumor cells (Fig. 1).

Recently, IAA/HRP was used for ADEPT of hematological malignancies 22. Hematopoietic cell lineages and primary tumor cells from patients with acute myeloid leukemia and chronic lymphocytic leukemia were targeted with HRP‐conjugated antibodies and then incubated with different concentrations of IAA. Although IAA/HRP caused apoptosis in hematopoietic tumors, this study was only performed *in vitro* due to a lack of biocompatible and well‐defined preparations of single HRP isoenzymes. Hitherto existing studies were performed with commercially available HRP which derives from plant and describes a mixture of isoenzymes with a heterogenous plant‐derived glycosylation pattern causing rapid clearance of HRP‐conjugates from the human body 28. Thus, the use of HRP in ADEPT and respective *in vivo* studies were limited.

In this study, we recombinantly produced the two single HRP isoenzymes C1A and A2A in the yeast *P. pastoris*. This yeast species has the tendency for hyperglycosylating recombinant proteins 29, which prevents medical applications due to the incompatibility of yeast‐derived surface glycans with the human body 30. Thus, we did not only use a *P. pastoris* benchmark strain for production, but also a strain where the *α*‐1,6‐mannosyltransferase (OCH1), responsible for hyperglycosylation in the yeast, was knocked out (Δoch1 strain; 31, 32). In total, we produced and biochemically characterized four enzyme variants (Table 1). We analyzed the efficiency of these enzyme variants to oxidize IAA and finally tested them in combination with IAA on human T24 bladder carcinoma and MDA‐MB‐231 breast carcinoma cells *in vitro*. To our knowledge this is the first study describing the use of recombinant HRP for *in vitro* cancer treatment.

**Table 1:** *Pichia pastoris* strains and horseradish peroxidase (HRP) isoenzymes used in this study

*P. pastoris* strain	HRP isoenzyme	Strain/enzyme designation
benchmark	C1A	bmC1A
	A2A	bmA2A
Δoch1	C1A	Δoch1C1A
	A2A	Δoch1A2A

## Material and Methods

### Strain generation

All strains in this study are based on the *P. pastoris* wild‐type strain CBS7435. The recombinant *P. pastoris* strains were provided by Prof. Anton Glieder (University of Technology, Graz, Austria). Strain generation is described in detail in our previous studies 31, 33.

### Bioreactor cultivation

The recombinant *P. pastoris* strains were cultivated in the controlled environment of a bioreactor. We performed dynamic batch cultivations with consecutive methanol pulses to determine strain physiological parameters which presented the basis for subsequent fed‐batch cultivations. We described this strategy of efficient bioprocess development for recombinant *P. pastoris* strains in several of our previous studies in detail 31, 32, 34, 35.

The dynamic batches where methanol was repeatedly pulsed at a concentration of 1% (*v*/*v*) at 20°C served the purpose of determining the maximum specific methanol uptake rate (*q*_s max MeOH_). For the subsequent fed‐batch cultivations, batch and uninduced fed‐batch were performed on glycerol at 30°C, followed by a methanol adaption pulse and a subsequent methanol fed‐batch at 20°C. According to the respective *q*_s max MeOH_, we controlled the feed rate corresponding to a *q*_s__MeOH_ of 0.70 mmol/g per hour for the benchmark strains and of 0.20 mmol/g per hour for the Δoch1 strains. By feeding at this low *q*_s_ we minimized the risk of overfeeding the cells. In all bioreactor cultivations we added either 10 *μ*mol/L hemin or 1 mmol/L Δ‐aminolevulinic acid as heme precursor 36.

### Protein purification

Cell‐free supernatants were diafiltrated in binding buffer (20 mmol/L NaOAc, 0.5 mol/L NaCl, pH 6.0). The diafiltrated protein solutions were loaded on a hydrophobic charge induction chromatography (HCIC) resin (MEP HyperCel, Pall, Vienna, Austria). The chromatographic runs were performed in flow‐through mode 31, 37, 38. The HCIC resin was equilibrated with 5 column volumes (CV) binding buffer. After a postload wash of 4 CV binding buffer, elution was performed as a one‐step gradient with elution buffer (50 mmol/L Tris, 1 mol/L NaCl, pH 8.0). All steps were performed at a flow rate of 55 cm/h. The efficiency of purification was evaluated by the purification factor (PF; eq. (1)) and the recovery yield of HRP activity in percentage (*R*%; eq. (2)). The suffixes “pre” and “post” indicate the respective values before and after the HCIC step.

(1) $$\text{PF}=\frac{\text{specific}\;{\text{activity}}_{\mathrm{post}}}{\text{specific}\;{\text{activity}}_{\mathrm{pre}}}$$

(2) $$R\%=\frac{\text{volumetric}\;{\text{activity}}_{\mathrm{post}}\times {\text{volume}}_{\mathrm{post}}}{\text{volumetric}\;{\text{activity}}_{\mathrm{pre}}\times {\text{volume}}_{\mathrm{pre}}}$$

### Biochemical enzyme characterization

The enzyme variants were biochemically characterized as described in our previous studies 38, 39. We included plant HRP in all the analyses for comparison. The measurements of the basic kinetic parameters maximum reaction rate (*v*_max_) and Michaelis constant (*K*_M_) were performed using 2,2′ azino‐bis‐3‐ethylbenz‐thiazoline‐6‐sulfonic acid (ABTS) and H_2_O_2_ as substrates. Changes in absorption were detected in a spectrophotometer (UV‐1601, Shimadzu Duisburg, Germany) at 420 nm and 30°C for 180 sec. The reaction mixture with a final volume of 1 mL contained 20 *μ*L enzyme preparation, 1 mmol/L H_2_O_2_ and varying ABTS concentrations (0.05–10 mmol/L) in 50 mmol/L potassium phosphate buffer (pH 6.5). The absorption curves were recorded (UVPC Optional Kinetics software, Shimadzu) and *v*_max_ and *K*_M_ were calculated with Sigma Plot (Systat Software Inc., Washington, Chicago, US).

Thermal stability of the enzyme variants was determined by measuring the residual catalytic activity after 2.5, 5, 10, 15, 30, 60, 90, and 120 min incubation at 60°C. We chose 60°C due to the presence of several comparable studies in literature 31, 38, 39, 40. Measurements were done at final concentrations of 1 mmol/L H_2_O_2_ and 10 mmol/L ABTS. For stability testing we used comparable enzyme concentrations: plant HRP 1.0 *μ*g/mL, bmC1A 1.4 *μ*g/mL, bmA2A 3.8 *μ*g/mL, Δoch1C1A 13.8 *μ*g/mL and Δoch1A2A 13.7 *μ*g/mL.

### Oxidative activation of IAA

The reaction rate of the different HRP variants with IAA was measured via stopped‐flow analysis. IAA, indole‐3‐carbinol, indole‐3‐aldehyde and plant HRP were obtained from Sigma‐Aldrich (Vienna, Austria). Enzyme concentrations were determined by measuring the absorbance at 402 nm (*ε *= 1.02 × 10^5^ L/mol/L per cm) 41. Hydrogen peroxide stock solution concentration was determined at 240 nm (*ε *= 39.4 L/mol/L per cm) 42. IAA stock solutions (3 mmol/L) were prepared fresh daily in 1% (*v*/*v*) ethanol with 50 mmol/L potassium phosphate buffer (pH 6.5). Measurements of reaction rates of HRP compound I with IAA were determined using a double mix stopped‐flow spectrophotometer (Hi‐tech SF‐61 DX2) at 25°C 43. HRP (1.54 *μ*mol/L) was mixed equimolar with H_2_O_2_ for 1 sec in the age‐loop to form compound I and then this was sequentially mixed with IAA to give a final concentration of HRP of 0.5 *μ*mol/L and IAA ranging from 25 to 150 *μ*mol/L. The exponential formation of compound II was monitored at 418 nm and kinetic traces were analyzed using Kinetic studio software (TgK Scientific, Bradford‐on‐Avon, UK). The reaction rate of compound I with IAA was determined from the nonlinear least squares fit slope of the observed rates (*k*_obs_) against IAA concentration.

To analyze the reaction products, IAA (100 *μ*mol/L) was placed in a glass vial with or without H_2_O_2_ (10 *μ*mol/L) and allowed to reach 37°C. The reaction was initiated by the addition of 15 nmol/L peroxidase. Samples were measured repeatedly for up to 6 h by HPLC analysis (Waters 2695 equipped with a photodiode array detector Waters 2996). Separation was achieved with a reversed phase RPB HPLC column (100 × 3.2 mm, 5 *μ*m; Hichrom) with a flow rate of 1 mL/min and a linear gradient comprising 5 mmol/L ammonium acetate buffer pH 5.1 and 75% (*v*/*v*) acetonitrile. Reaction products were separated with a gradient of 15–75% (*v*/*v*) acetonitrile. Products were identified by comparison with commercially available standards (Sigma‐Aldrich). Skatoyl hydroperoxide (IAA‐OOH), oxindole‐3‐carbinol (OXI) and 3‐methylene‐2‐oxindole (MOI) were identified by comparison with previously recorded spectra 41.

### *In vitro* cytotoxicity study

Human T24 bladder and MDA‐MB‐231 breast carcinoma cells were obtained from the American Type Culture Collection (ATCC; Manassas, VA) and used within the first eight passages from the supplier. Both cell lines were maintained in Dulbecco's Modified Eagle medium (DMEM, Life Technologies, Vienna, Austria) supplemented with 10% fetal calf serum (FCS), 100 U/mL penicillin (Sigma) and 100 *μ*g/mL streptomycin (Sigma) in 75 cm² rectangular canted neck cell culture flasks. The cells were maintained in a humidified incubator at 37°C. Trypsin was used as the detaching agent for processing the cells after each growth phase. Determination of the cell number was performed by staining with Trypan blue and counting the viable cells with a Countess cell counter (Invitrogen, Vienna, Austria).

After seeding in 96‐well plates with 5,000 T24 cells per well and 10,000 MDA‐MB‐231 cells per well, the cells were allowed to attach for 3 h before administration of the prodrug and different enzyme variants. The attached cells in the 96 well plates were treated with a final concentration of 0.1, 0.5, 1.0, 1.5, and 2 mmol/L IAA and 1.2 *μ*g/mL HRP in Roswell Park Memorial Institute medium (RPMI‐1640 medium without phenol red) per well, according to prior studies 4, 5, 24. IAA solutions and HRP dilutions were prepared fresh daily. The incubation of the cells with different enzyme/prodrug combinations, enzymes only or IAA solution only, as negative control, was done at 37°C for 72 h. Additionally, tests with the commercial cytostatic drug cisplatin (Sigma) were performed with final concentrations ranging from 0.1–100 *μ*mol/L cisplatin per well to act as positive controls for cytotoxicity. After 72 h of drug exposure, freshly prepared 3‐(4,5‐dimethylthiazol‐2‐yl)‐2,5‐diphenyltetrazolium bromide (MTT) solution (0.5 mg/mL) was added to each well and incubated for 3 h to measure the metabolic activity of the cancer cells after treatment, as an indication of cell viability. After addition of solubilization solution (89% (*v*/*v*) 2‐propanol, 10% Triton X‐100, 0.1 mol/L HCl) the violet formazan crystals were dissolved and the absorbance was measured at 570 nm in a spectrometer (1420 Multilabel counter, Perkin Elmer).

## Results and Discussion

### Bioreactor cultivation

We performed dynamic batch cultivations to determine *q*_s max MeOH_ of the recombinant benchmark and Δoch1 strains. As shown in Table 2, *q*_s max MeOH_ values for the benchmark strains were higher than for the Δoch1 strains. In analogy to our previous studies, we observed that the Δoch1 strains lost metabolic activity over time 31, 32. Thus, to avoid the risk of overfeeding the cells, we followed the same strategy of feeding the recombinant Δoch1 strains at a *q*_s_ of only 0.2 mmol/g/h in the subsequent fed‐batch cultivations as we have done previously 32. After 60 h of induction we obtained around 200 mg total extracellular protein per liter cultivation broth in all cultivations.

**Table 2:** Maximum specific methanol uptake rates of the single recombinant *Pichia pastoris* strains determined in dynamic batch cultivations with three consecutive 1% methanol pulses at 20°C

*P. pastoris* strain	*q*_*s* max MeOH_ (mmol/g per h)
bmC1A	0.93
bmA2A	0.95
Δoch1C1A	0.75
Δoch1A2A	0.81

### Protein purification

The different HRP variants were purified from cell‐free cultivation broth by a 1‐step HCIC approach in flow‐through mode (Table 3).

**Table 3:** Hydrophobic charge induction chromatography (HCIC) purification of recombinant horseradish peroxidase (HRP) variants. The specific activity before and after HCIC, the overall purification factor (PF) and the recovery of HRP activity in the flow‐through in percentage (*R*%) are shown

Enzyme variant	Spec. activity before HCIC (U/mg)	Spec. activity after HCIC (U/mg)	PF	*R*%
bmC1A	244	1048	4.3	95.7
bmA2A	106	2086	19.7	98.5
Δoch1C1A	28	77	2.8	93.2
Δoch1A2A	50	927	18.5	94.8

As shown in Table 3, the purification worked more efficiently for HRP A2A than for HRP C1A which is in agreement with our previous observations 38. The reduced specific activities of enzyme preparations from the Δoch1 strains compared to benchmark strains can be ascribed to both an increased amount of impurities due to increased cell lysis of the Δoch1 strain and a reduced catalytic activity of HRP due to reduced surface glycans 40. However, compared to commercially available HRP isolated from plant with a specific activity of 1000 U/mg (Sigma‐Aldrich, P6782‐100MG) the purity of the final enzyme preparations was satisfactory.

### Biochemical enzyme characterization

In analogy to previous studies, the kinetic constants for ABTS/H_2_O_2_ and the thermal stability of the purified HRP variants were determined (Table 4; 31, 38, 39, 40).

**Table 4:** Kinetic constants for the substrates ABTS/H_2_O_2_ and thermal stability of commercial plant horseradish peroxidase (HRP) and different HRP variants

Enzyme variant	*K*_M_(mmol/L)	*v*_max_(mmol/L per sec)	*τ*_½_ 60°C(min)
Plant HRP	1.86 ± 0.33	517 ± 28.4	9.1
bmC1A	1.50 ± 0.20	153 ± 5.72	33.8
bmA2A	1.90 ± 0.25	238 ± 11.3	6.1
Δoch1C1A	1.56 ± 0.21	26.8 ± 1.26	29.0
Δoch1A2A	2.46 ± 0.34	98.3 ± 2.37	3.8

As shown in Table 4, glycosylation did not alter substrate affinity, but greatly affected catalytic activity. The presence of shorter Man8 surface glycans 31 might increase biocompatibility as it resembles an intermediate of human protein glycosylation but comes at cost of enzyme activity with ABTS/H_2_O_2_. Also thermal stability of enzyme variants from the Δoch1 strain was reduced compared to their hyperglycosylated counterparts, but was comparable to the stability of plant HRP. These results are in good agreement with previous observations 40. However, since these analyses did not give an indication for the activity of the HRP variants with IAA and thus their potential usefulness in cancer treatment, respective experiments were conducted.

### Oxidative activation of IAA

**Figure 2:**
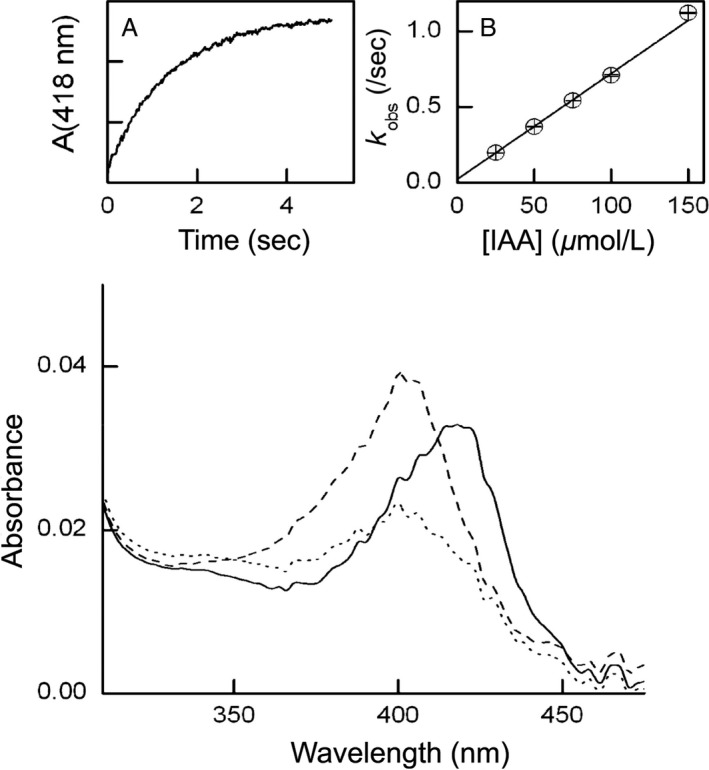
Absorption spectra of bmA2A in the ferric state (~ 0.5 *μ*mol/L, dashed line), after addition of equimolar H_2_O_2_ (compound I, dotted line) and after subsequent addition of 10 *μ*mol/L indole‐3‐acetic acid (IAA). Insert A: observed increase of absorbance at 418 nm on reaction of bmA2A compound I with 100 *μ*mol/L IAA. Insert B: linear dependence of the observed rate of build‐up of bmA2A compound II at 418 nm as a function of IAA concentration; error bars represent the standard deviation of three measurements. The experiments were carried out in 50 mmol/L potassium phosphate buffer at pH 6.5 and 25°C.

Stopped‐flow analysis was used to measure the turnover rate of compound I to II at 418 nm (Fig. 2) and thus depict the reaction of each HRP variant with IAA at 25°C (Table 5). We adjusted the temperature to 25°C due to technical limitations. IAA reacts with plant HRP at a rate constant of 9.6 × 10^3^ dm^3^/mol per sec. All recombinant HRP variants showed comparable reaction rates. Enzyme bmC1A oxidized IAA even twofold faster than plant HRP when comparing protein concentration equivalents. Unfortunately the reaction rate of enzyme Δoch1C1A could not be determined reliably by stopped‐flow analysis due to the formation of precipitates during reaction.

**Table 5:** Reaction rate constants of horseradish peroxidase (HRP) enzyme variants with indole‐3‐acetic acid (IAA) in 50 mmol/L phosphate buffer at pH 6.5 and 25°C

Enzyme variant	*k* (cpd I) (dm^3^/mol per sec)^a^	*k* (cpd I) (dm^3^ /mol per sec)/0.1 mg/mL protein^b^
Plant HRP	(9.6 ± 0.4) × 10^3^	(2.9 ± 0.1) × 10^4^
bmC1A	(1.1 ± 0.01) × 10^4^	(5.0 ± 0.04) × 10^4^
bmA2A	(7.4 ± 0.1) × 10^3^	(2.2 ± 0.03) × 10^4^
Δoch1C1A	n.d.	n.d.
Δoch1A2A	(6.9 ± 0.3) × 10^3^	(1.6 ± 0.1) × 10^4^

n.d., not determined.

a Rate constant determined using approximately 0.5 *μ*mol/L HRP and 0.5 *μ*mol/L H_2_O_2_.

b Rate constant normalized to 0.1 mg/mL protein.

**Figure 3:**
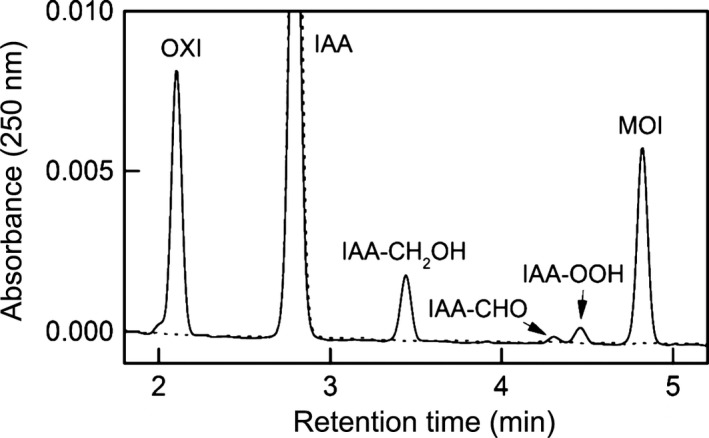
Chromatogram showing the reaction of indole‐3‐acetic acid (IAA) (100 *μ*mol/L) with plant horseradish peroxidase (HRP) (1.4 U) after 0 min (dotted line) and 66 min (solid line) at 37°C in 50 mmol/L potassium phosphate buffer (pH 6.5). Peaks were observed for oxindole‐3‐carbinol (OXI), IAA, indole‐3‐carbinol (IAA‐CH_2_ OH), indole‐3‐aldehyde (IAA‐CHO), skatoyl hydroperoxide (IAA‐OOH), and 3‐methylene‐2‐oxindole (MOI).

The turnover of IAA with each of the HRP variants with and without the addition of 10 *μ*mol/L H_2_O_2_ was also analyzed by HPLC at 37°C. We used 37°C to mimic the temperature in the human body. Reaction products were identified by comparison with commercially available standards and previously reported spectra 41. All HRP variants gave comparable reaction products. A typical chromatogram is exemplarily shown for plant HRP at 37°C in Figure 3.

**Figure 4:**
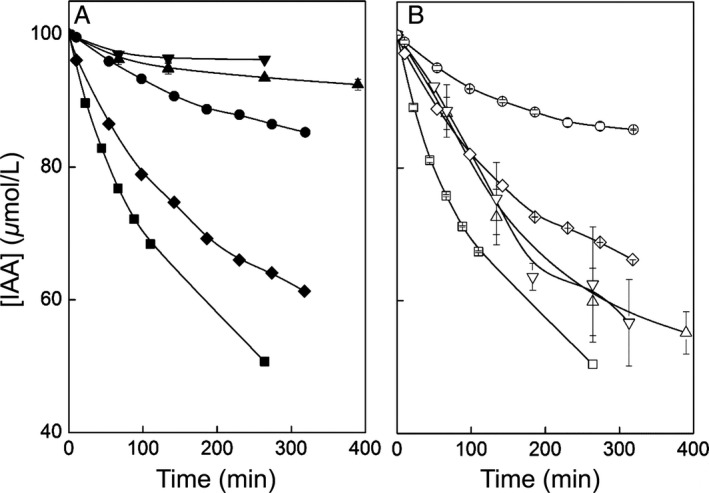
Turnover of indole‐3‐acetic acid (IAA) (100 *μ*mol/L) with different horseradish peroxidase (HRP) variants. Squares, HRP plant peroxidase (1.4 U); diamonds, bmC1A (0.7 U); circles, Δoch1C1A (0.7 U); triangles up, bmA2A (3.5 U); triangles down, Δoch1A2A (3.9 U); all in the absence (A, solid symbols) or presence (B, open symbols) of 10 *μ*mol/L H_2_O_2_. All reactions were carried out in 50 mmol/L potassium phosphate buffer at pH 6.5 and 37°C.

By regular sampling, we were able to depict the reaction rates of plant HRP and the HRP variants with IAA at 37°C. As shown in Figure 4, all HRP variants oxidized IAA in the presence of 10 *μ*mol/L H_2_O_2_. Plant HRP, bmC1A, and Δoch1C1A turned over IAA at a comparable rate with and without H_2_O_2_, which is a prerequisite for ADEPT. Surprisingly, enzymes bmA2A and Δoch1A2A were ineffective at metabolizing IAA in the absence of H_2_O_2_. We conclude that oxidation of IAA in the absence of H_2_O_2_ is HRP isoenzyme specific. However, both isoenzymes metabolized IAA in the presence of H_2_O_2_ also with reduced surface glycosylation (Fig. 4).

**Figure 5:**
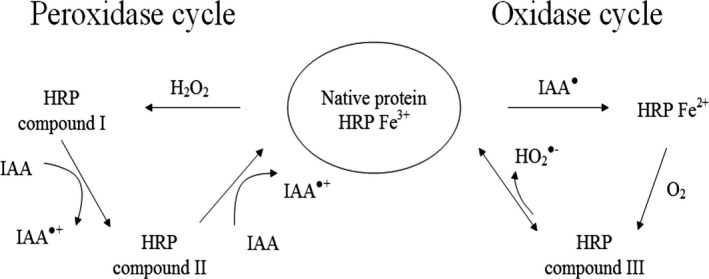
Interconversion of horseradish peroxidase (HRP) enzyme states through the peroxidase and oxidase pathway. Figure adapted from 46.

Plant HRP can oxidize IAA either via a H_2_O_2_‐dependent pathway (peroxidase cycle) or a H_2_O_2_‐independent but oxygen‐dependent mechanism (oxidase cycle) (Fig. 5; 41, 44, 45). Although the mechanism by which HRP reacts with IAA and the dependence on H_2_O_2_ is still not fully understood, there is a consensus that ferrous peroxidase and HRP compound III are involved in some way 41, 45, 46, 47, 48, 49. As HRP C1A is able to oxidize IAA in the absence of H_2_O_2_, skatoyl hydroperoxide may be able to replace H_2_O_2_ during the peroxidase cycle 44. The extent to which the oxidase and peroxidase pathways are utilized may depend upon the relative concentration ratios of HRP to IAA. When HRP concentrations exceed 2 × 10^−7^ mol/L and IAA <0.5 mmol/L the conventional peroxidase pathway is followed but when the ratio is lower (HRP <4 × 10^−8^ mol/L and IAA >50 *μ*mol/L) a mechanism involving molecular oxygen, ferrous enzyme and compound III (Fe^3+^‐O_2_˙^−^) is utilized 45. The production of superoxide through the oxidase pathway may be an additional cause of IAA/HRP‐induced cell toxicity which could potentially be harnessed for therapy 50. Different HRP isoenzymes were shown to have differing activities in oxidase and peroxidase reactions 51. Thus, it seems reasonable that HRP C1A and HRP A2A differ in their reactivity toward IAA. Since we found that HRP A2A is largely dependent upon H_2_O_2_ for the reaction with IAA, we speculate that this isoenzyme has an inefficient oxidase pathway.

In summary, we showed that both isoenzymes oxidized IAA independent of surface glycosylation but that HRP A2A cannot be used in cancer therapy due to its dependence upon H_2_O_2_. Thus, we only used HRP C1A variants for subsequent *in vitro* cytotoxicity studies.

### *In vitro* cytotoxicity study

**Figure 6:**
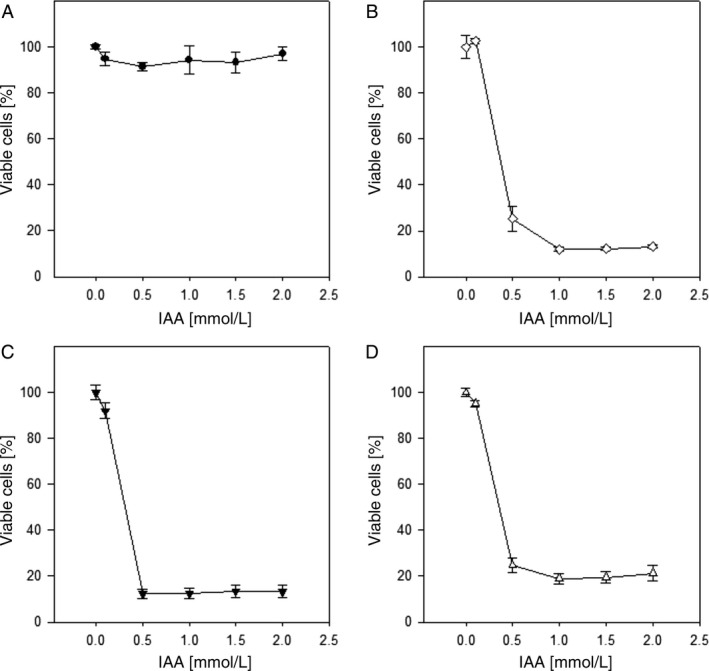
Cytotoxicity of enzyme/prodrug treatment on T24 bladder carcinoma cells after 72 h at a constant horseradish peroxidase (HRP) concentration of 1.2 *μ*g/mL and varying indole‐3‐acetic acid (IAA) prodrug concentrations. The data are weighted means of three independent experiments (triplicate samples). The error bars represent the standard error. (A) no HRP; (B) bmC1A; (C) Δoch1C1A; (D) commercial plant HRP.

**Figure 7:**
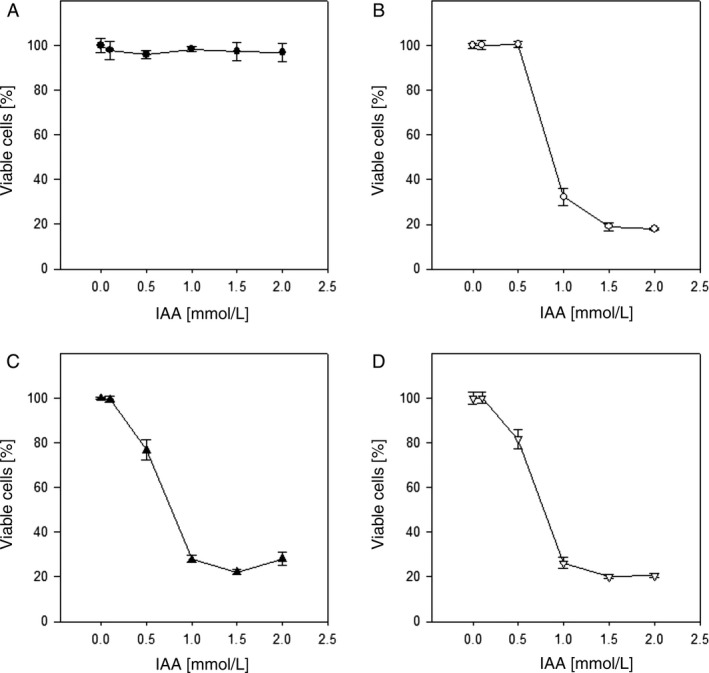
Cytotoxicity of enzyme/prodrug treatment on MDA‐MB‐231 breast carcinoma cells after 72 h at a constant horseradish peroxidase (HRP) concentration of 1.2 *μ*g/mL and varying indole‐3‐acetic acid (IAA) prodrug concentrations. The data are weighted means of three independent experiments (triplicate samples). The error bars represent the standard error. (A) no HRP; (B) bmC1A; (C) Δoch1C1A; (D) commercial plant HRP.

We used commercial plant HRP, bmC1A, and Δoch1C1A in combination with different concentrations of IAA for *in vitro* cytotoxicity studies on human T24 bladder and MDA‐MB‐231 breast carcinoma cells. A HRP concentration of 1.2 *μ*g/mL was used according to prior studies 4, 5, 24 and several IAA concentrations ranging from 0.1 to 2 mmol/L were tested. After 72 h, MTT assays revealed cytotoxic effects of the different enzyme/prodrug combinations (Figs. 6 and 7). The administration of HRP variants alone as well as IAA alone acted as negative controls, and showed no toxicities under the conditions tested (Figs. 6A and 7A). As expected, cisplatin reduced viability significantly in both cell lines, with Ic50 values of 1.4 and 14 *μ*mol/L for T24 and MDA‐MB‐231, respectively. Once IAA was applied in combination with HRP, significant cytotoxic effects were observed. The viability of T24 bladder carcinoma cells was reduced to less than 20% in the presence of 1.2 *μ*g/mL HRP and a concentration between 100 and 500 *μ*mol/L IAA (Fig. 6B–D).

The same trend was observed for MDA‐MB‐231 breast carcinoma cells, with 25% cells remaining viable (Fig. 7). The shift to increased resistance to IAA compared to T24 cells can be explained by tumor cell line‐specific properties.

This outcome validates the results from previous studies describing the successful treatment of cancer cells with plant HRP as part of ADEPT 5, 24. However, we are the first ones to show this cytotoxic effect using a range of different recombinant HRP variants. In Table 6 the Ic50 values of the different HRP variants at a concentration of 1.2 *μ*g/mL are summarized. Commercial plant HRP needed the highest concentration of IAA to exhibit cytotoxic effects, whereas recombinant HRP variants needed less IAA. This can be explained by the fact that plant HRP describes a mixture of isoenzymes, with some of them being unable to metabolize IAA (e.g., HRP A2A, as shown in this study). Interestingly, the combination of Δoch1C1A with IAA showed the highest cytotoxic effect. Thus, we propose to specifically produce HRP C1A with reduced surface glycosylation and use it in combination with IAA in ADEPT as a novel strategy for targeted cancer treatment.

**Table 6:** Ic50 values of indole‐3‐acetic acid (IAA) for T24 bladder and MDA‐MB‐231 breast carcinoma cell lines at a concentration of 1.2 *μ*g/mL horseradish peroxidase (HRP)

	T24 bladder carcinoma cells	MDA‐MB‐231 breast carcinoma cells
Enzyme variant(1.2 *μ*g/mL)	Ic50 for IAA (mmol/L)	Ic50 for IAA (mmol/L)
Plant HRP	0.473 (±0.002)	0.877 (±0.08)
bmC1A	0.224 (±0.02)	0.628 (±0.01)
Δoch1C1A	0.115 (±0.001)	0.570 (±0.04)

Summarizing we demonstrated the power of recombinant, glyco‐engineered HRP isoenzyme C1A and IAA for targeted cancer treatment. Although reduced surface glycoslyation changed biochemical properties, glyco‐engineered HRP variants efficiently metabolized IAA. The use of a humanized yeast strain as expression platform and protein engineering for boosted enzyme activity might allow the increased use of IAA/HRP in *in vivo* ADEPT in the future.

## Conflict of Interest

The authors declare no conflict of interest.
